# What We Owe to Experiments on Animals

**Published:** 1904-01-23

**Authors:** Stephen Paget


### HOSPITAL CLINICS AND MEDICAL PROGRESS.

### WHAT WE OWE TO EXPERIMENTS ON ANIMALS.

By STEPHEN PAGET.

### II.—PATHOLOGY, BACTERIOLOGY, AND THERAPEUTICS.

(*Continued from page* 273.)

### VII.—CHOLERA.

The cholera bacillus was discovered by Koch in 1883. Then came a long period of controversy, which lasted till the Hamburg epidemic of 1892. The laboratory work, and all the work, involved many difficulties and some danger:—

"In order to prove that this *vibrio* is the cause of Asiatic cholera, several tests upon themselves have been voluntarily made by investigators in laboratories. These were carried out in Munich and in Paris. The results to the experimenters were sufficiently severe to indicate positively the pathogenic character of the spirillum, and its capacity to produce cholera-like infections."—(*Stedman's Twentieth Century Practice*, vol. xix. 1900.).

Haffkine, Pasteur's pupil, began in 1889 to study how to immunise animals against the disease. Other countries were at work over the same problem. By 1893, the immunisation of animals was proved and exactly measured. "The physiological and pathological effect on man was then studied on some sixty persons, mostly medical and scientific men interested in the solution of the problem. The effect was found to be harmless to health. The next step was to transfer the operations to the East."—(*Haffkine's Report to Indian Government.* 1895.).

For the next two-and-a-half years (April 1893—July 1895) Haffkine was in India, having turned up at Calcutta in March 1893, a veritable knight-errant, with a blessing from our Government and half-a-dozen inoculated rabbits in baskets. In 1895, he was invalided home, having administered more than 70,000 injections to 42,179 persons—officers, soldiers, native soldiers, Europeans, Eurasians, and 31,056 natives—*without having to record a single instance of mishap or accident of any description caused by our vaccines.* His report to the Government of India (1895) and his London lecture (see *Brit. Med. Jour.*, December 21st, 1895) must be read here; and Dr. Simpson's 1896 *Report* (see *Lancet*, August 8th, 1896). See also the thirtieth *Annual Report of the Sanitary Commissioner for Bengal*, 1897, and the *Indian Medical Gazette*, May, 1898, and September, 1901.

Certainly, among 300,000,000 people, these inoculations can no more save India from cholera than Government relief works can save her from famine. We must take what results are to be had, and wait for more. Dr. Simpson's *Report* (1896) gives the results, up to 1896, of all those inoculations which allowed an exact comparison of the respective powers of resistance against cholera of inoculated and non-inoculated persons. A full account of this report is given in "Experiments on Animals" (John Murray, 1903). It deals with eight groups of persons living and working together, some inoculated, some non-inoculated:—

1. *Calcutta* (1894–1896).—No account is taken of cases of cholera in houses where nobody was inoculated. No account is taken of houses whose inmates were inoculated and no cases of cholera occurred. Those houses alone are taken into account where cholera occurred in spite of inoculations. There were 77 of these houses. In 1, everybody had been inoculated. In 76, inoculated and non-inoculated were living together. Of these 76 houses 6 are excluded, because they contained so few inoculated that the escape of these inoculated might be due, as it were, to chance. That leaves 70 houses. The results in these 70 houses were:—Of 654 non-inoculated, 71 died=10·86 per cent.; of 402 inoculated, 12 died=2·99 per cent.

2. *East India Regiment, Lucknow* (1893).—The results here were disappointing, from reasons that are fully explained in the Report. Of 640 non-inoculated, 79 died=12·34 per cent. Of 133 inoculated, 13 died=9·7 per cent.

3. *Gaya Jail* (1894).—Of 202 non-inoculated, 10 died=4·95 per cent. Of 207 inoculated, 5 died =2·41 per cent.

4. *Assam-Burmah Railway* (? 1895).—This was a group of 355 coolies and native soldiers, working under a surveying party. The majority were inoculated. Of 159 non-inoculated, 30 died=18·8 per cent. Of 196 inoculated, 4 died=2·04 per cent.

5. *Durbhanga Jail* (1896).—The figures here are based on a daily average, as the population of the jail shifted a little. Of a daily average of 99 non-inoculated, 11 died=11·11 per cent. Of 110 inoculated, 3 died=2·73 per cent.

6 and 7. In a coolie camp at *Bilaspur*, of 100 non-inoculated, 5 died=5 per cent. Of 150 inoculated, 1 died=0·6 per cent. In *Serampur*, among the general population, of 51 non-inoculated, 3 died=5·5 per cent. Of 42 inoculated, 1 died= 2·4 per cent.

8. *Cachar Tea Gardens* (1895).—These results are excellent, and deal with large figures. Of 2,976 non-inoculated, 60 died=2 per cent. Of 2,381 inoculated, 4 died=0·16 per cent.; and 1 of these 4 deaths was doubtful. We may fairly reckon that the non-inoculated, living and working and feeding side by side with the inoculated, lost 15 lives to their 1.

---

Haffkine, in his comment on these figures, says—"In the Gaya jail, the inoculations were *for the first time* applied *in a prevalent epidemic*, and *very weak doses* of a relatively weak vaccine were used. Far higher results have been obtained by an application of stronger doses. In the *bustees* situated round the tanks in Calcutta, where cholera exists in a permanent state, the disease occurred in 36 houses with inoculated people. In each of these houses there was one part of the family inoculated and another not. The observations were continued for 459 days, with the following results. During the first period of 5 days, subsequent to the inoculation with *first* vaccine, cholera occurred in 8 houses: 75 non-inoculated had 5 cases with 3 deaths; 52 inoculated had 3 cases with 3 deaths. During the second period of 5 days, subsequent to the *second* inoculation, cholera occurred in 2 houses: 8 non-inoculated had 2 cases with 2 deaths; 17 inoculated had no cases. *After the 10 days necessary for the preventive treatment had expired, and up to the 459th day, the disease visited 26 houses: 273 non-inoculated had 38 cases with 34 deaths; 137 inoculated had 1 case with 1 death, in a child that had not been brought up for the second inoculation.*"

It would be hard to find a better instance of successful preventive treatment than these 36 houses or huts, crowded with natives, and huddled round a common water-supply: the two groups of lives, alike in everything—in food and water and air and habits and environment—the one group protected, the other not protected. Then the cholera went round the huts, and picked out 38 cases with 34 deaths among the non-protected, and 1 case among the protected; and that 1 case had been only half-protected.

But let us take larger and more recent figures, from the Report for 1900 of the Sanitary Commissioner for Bengal. They are quoted in the *Indian Medical Gazette*, September, 1901, which says:—"We are glad to see from a paragraph in the Report of the Sanitary Commissioner for Bengal (Major H. J. Dyson, I.M.S., F.R.C.S.), that an increased number of anti-cholera inoculations were performed during the year 1900. Assistant-Surgeon G. C. Mukerjee, who was in charge of this work, reports that in the Puralia coolie depôt no less than 13,291 persons were inoculated against cholera, including over 1,000 children. All these cases of inoculation were among labour emigrants proceeding to the tea-gardens of Assam and Cachar. *The employers of labour are beginning to realise the value of cholera inoculation.* It is unfortunately not always easy, or even possible, to follow up the after-history of persons inoculated, but Major Dyson has quoted a table, received from the Superintendent of Emigration, which shows the number of cases among the inoculated and the non-inoculated at Goalundo. From this table it is seen that out of 1,527 non-inoculated coolies who passed through Goalundo, 33, or 2·09 per cent., got cholera; whereas of 873 inoculated coolies only 2, or 0·2 per cent., were attacked by the disease; that is, the unprotected suffered about ten times as much as the inoculated."

There is a sting in that sentence, *The employers of labour are beginning to realise the value of cholera inoculation.* But nothing will tell more forcibly on men's minds than these inoculations among factory hands, mill hands, railway coolies, and jail-birds. Given a thousand natives, living and sleeping and working side by side: the same water-supply and food and discipline for all: and to each one of them, for fear of cholera, let the preventive treatment be offered. Some will accept it; some will take their chance. Then, in this close and guarded crowd of monotonous lives, when the cholera comes, it will select its cases, so that *the employer of labour* shall be compelled to see the meaning of Haffkine's work.

In the book, "Experiments on Animals," the following sentence was added by Lord Lister himself: "Another most important result of the discovery of the cholera-bacillus is its use in diagnosis. For example, if a case of suspected cholera is landed at a British port, the sanitary authority at once takes steps to ascertain whether the specific microbe is present; and, according to the answer given by bacteriology, either allows the patient to proceed on his journey, or adopts measures of isolation to prevent the spread of the disease to others. Thus, thanks to the insular position of Great Britain, this dreadful disease has for many years been prevented from invading her population."

(*To be continued.*)

